# Genome-wide phenotypic RNAi screen in the *Drosophila* wing: phenotypic description of functional classes

**DOI:** 10.1093/g3journal/jkab349

**Published:** 2021-10-02

**Authors:** Ana López-Varea, Patricia Vega-Cuesta, Ana Ruiz-Gómez, Cristina M Ostalé, Cristina Molnar, Covadonga F Hevia, Mercedes Martín, Maria F Organista, Jesus de Celis, Joaquín Culí, Nuria Esteban, Jose F de Celis

**Affiliations:** 1 Centro de Biología Molecular “Severo Ochoa,” CSIC and Universidad Autónoma de Madrid, Madrid 28049, Spain; 2 IRB Barcelona, Barcelona 08028, Spain

**Keywords:** phenotype, wing, screen, RNAi

## Abstract

The *Drosophila* genome contains approximately 14,000 protein-coding genes encoding all the necessary information to sustain cellular physiology, tissue organization, organism development, and behavior. In this manuscript, we describe in some detail the phenotypes in the adult fly wing generated after knockdown of approximately 80% of *Drosophila* genes. We combined this phenotypic description with a comprehensive molecular classification of the *Drosophila* proteins into classes that summarize the main expected or known biochemical/functional aspect of each protein. This information, combined with mRNA expression levels and *in situ* expression patterns, provides a simplified atlas of the *Drosophila* genome, from housekeeping proteins to the components of the signaling pathways directing wing development, that might help to further understand the contribution of each gene group to wing formation.

## Introduction

Large-scale genetic screens are instrumental to identify the set of genes affecting a particular process (St Johnston 2002). In addition, the analysis of mutant phenotypes in individual tissues helps dissecting the mechanisms participating in its development (Molnar *et al.* 2006; Cruz *et al.* 2009). Sequencing and annotation of the *Drosophila* genome (Adams *et al.* 2000), in combination with the development of techniques to generate loss-of-function conditions using RNA interference now allow global descriptions of the phenotypic landscape, providing key information to understand the functional contribution of each gene or gene group to tissue development (Schnorrer *et al.* 2010; Valakh *et al.* 2012; Perkins *et al.* 2015; Ostalé *et al.* 2018).

The *Drosophila* wing has been the subject of a variety of genetic screens aiming to define genes affecting its growth and the patterning of structures such as the wing margin or the wing veins (Bjorklund *et al.* 2006; Molnar *et al.* 2006; Bejarano *et al.* 2008; Cruz *et al.* 2009). The wing develops from an epithelial tissue, the wing imaginal disc. The development of the wing disc involves regulated cell proliferation that drives the tissue from an embryonic primordium of 40 cells to the mature disc composed of approximately 50,000 cells (Beira and Paro 2016; Ostalé *et al.* 2018). In addition, the development of the disc involves a variety of cellular operations that are common to other multicellular developmental processes. They include the generation of gene expression domains underlying pattern formation and cell differentiation by complex networks of interactions between transcription factors and signaling pathways (Ostalé *et al.* 2018). The wing disc generates during metamorphosis a two-layered flat epithelial structure in which cells differentiate into cuticle or sensory organs in characteristic patterns. In this manner, the adult wing provides a readout of the cellular and molecular mechanisms underlying the development of the wing imaginal disc.

The understanding of mutant phenotypes is critical to identify the function of a gene. In classical genetics, a mutant phenotype precedes the knowledge of the biochemical characteristic of the protein encoded by the affected gene. Consequently, “gene functions” are assigned without any relation to the molecular function of the corresponding protein (St Johnston 2002; Kaufman 2017). However, since the sequencing and annotation of the *Drosophila* genome, we have access to relevant information about the biochemical function of gene products before any genetic data are available. Thus, the molecular annotation of the genome allows the correlation between mutant phenotypes and likely biochemical functions.

In order to correlate mutant phenotypes and biochemical functions, it is necessary to compile the available information about a protein into informative terms. The most complete source of information about gene function is the gene ontology (GO) database, which assigns to each gene product a set of definitions related to its molecular function, subcellular localization, and biological function (Ashburner *et al.* 2000). GO annotations allow enrichment analysis on gene sets, identifying the terms under- and over-represented in the set. As such, the use of GO enrichments is fundamental to extract meaningful biological information from data sets obtained in, for example, differential gene expression experiments or genetic screens. In the GO database, each gene is described by a collection of identifiers, the number of which depends on the current knowledge about the gene (Ashburner *et al.* 2000). For example, for a gene extensively studied such as *Notch*, GO assigns 130 terms. However, many of these terms are related to the multiple development processes where the function of Notch is required, and only two terms refer to its participation in a signaling pathway. Furthermore, if we compare the terms assigned to *Delta* (Dl; 61 terms) and *Suppressor of Hairless* [Su(H); 30 terms], two other components of the Notch signaling pathway, with those of Notch, we find only 4 coincidences from 221 aggregated terms. Most terms are either unique (97) or shared by two genes (57). For this reason, we generate a simplified nomenclature compacting the different GO descriptions into a set of terms encompassing the key biological aspect of a gene product. This classification can be used for enrichment analysis focusing on the most relevant aspect of each gene function.

**Figure 1:**
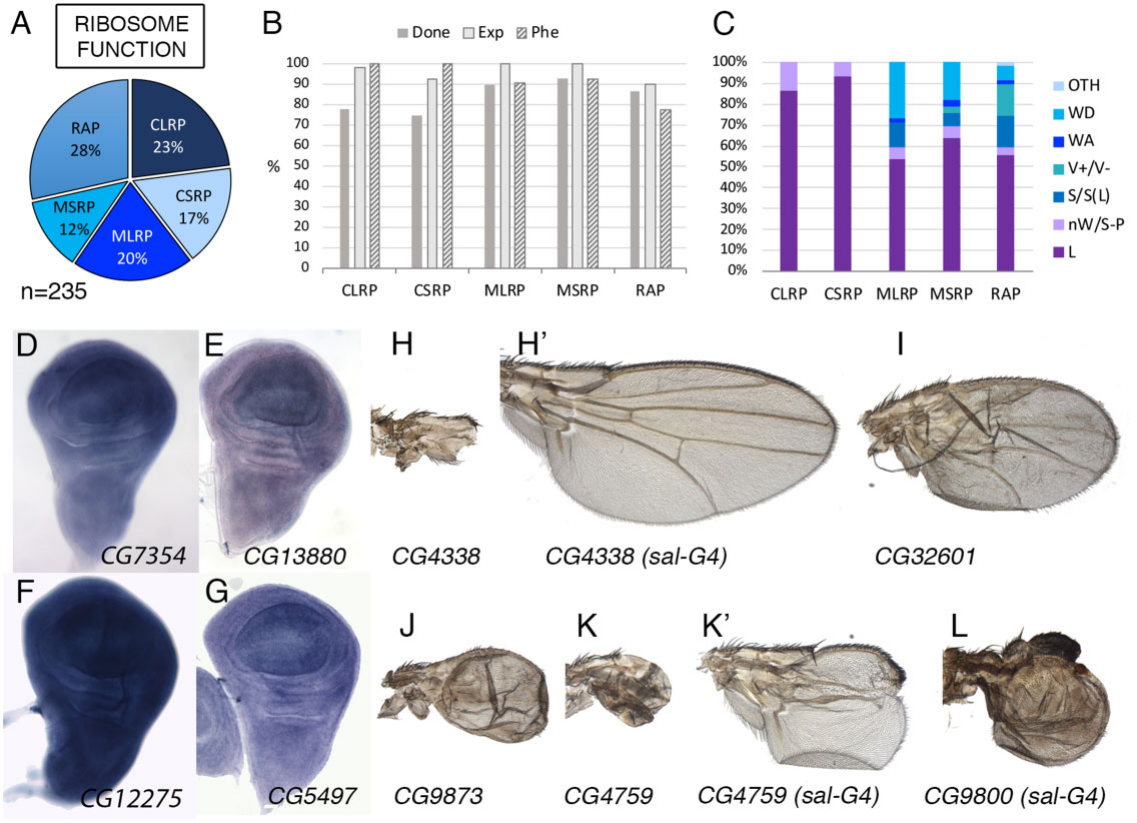
Ribosomal class. (A) Distribution of the 235 genes included in the ribosomal class. Cytoplasmic large and small ribosomal subunits (CLRP and CSRP), Mitochondrial large and small ribosomal subunits (MLRP and MSRP) and Ribosomal-associated proteins (RAP). (B) Percentage of genes of the ribosomal class groups for which we tested its knockdown phenotype (Done; dark gray column), genes expressed in the wing disc (Exp; light gray column), and genes with a lethal or visible phenotype in knockdown conditions (Phe; striped column). (C) Frequency of mutant phenotypes observed in the CLRP, CSRP, MLRP, MSRP, and RAP groups: L (lethal), nW (failure to form the wing), S-P (changes in the size of the wing and relative positions of the veins), S (size of the wing), V (including ectopic or thicker veins and loss of veins), WA (failures in the adhesion between the dorsal and ventral wing surfaces), and WD (altered cuticular differentiation). The phenotypes WS (shape of the wing), WM (defects in the wing margin), CD (changes in cell size or trichome differentiation), WP (changes in wing pigmentation), and Q (differentiation of ectopic bristles in the wing and loss of bristles in the wing margin) were grouped as “Others” (OTH). (D–G) Expression pattern in the wing disc of the genes *CG7354* (*mRpS26*), *CG13880* (*mRpL17*), *CG12275* (*RpS10a*), and *CG5497* (*mRpS28*), showing robust and generalized expression. *In situ* hybridizations pictures correspond to experiments published as supplementary material in Molnar *et al.* (2012), Organista *et al.* (2015), and Hevia *et al.* (2017). (H–H’) Adult wings of *UAS-Dicer2/+; nub-Gal4/UAS-CG4338-RNAi* (H) and *UAS-Dicer2/+; sal^EPv^-Gal4/UAS-CG4338-RNAi*, showing the transition from “nW” to “S” phenotype. (I, J) Adult wings of *UAS-Dicer2/+; nub-Gal4/UAS-betaNACtes3-RNAi* (I) and *UAS-Dicer2/+; nub-Gal4/UAS-RpL37b-RNAi*. (K–K’) Adult wings of *UAS-Dicer2/+; nub-Gal4/UAS-RpL27-RNAi* (K) and *UAS-Dicer2/+; sal^EPv^-Gal4/UAS-RpL27-RNAi* (K’), showing the more frequently observed transition from a “nW” to a “S-P” phenotype. (L) Adult wing of *UAS-Dicer2/+; sal^EPv^-Gal4/UAS-RpS18-RNAi* genotype showing the more frequently found class of S-P phenotype.

The adult wing in flies raised under standard conditions has a constant size, morphology, and pattern of differentiated elements. Changes in any of these characteristics are the constituents of each mutant phenotype, and they manifest in different combinations in each mutant wing. For the description of mutant phenotypes, we develop a simplified terminology reflecting the morphological changes observed in each mutant wing such as its size [S and S(L)]), wing size and patterning of veins (S-P), differentiation of veins (V− and V+), defects in the wing margin (WM), defects in dorsoventral wing surfaces adhesion (WA), defects in wing pigmentation (WP), changes in trichome size, number, polarity, or spacing (cell differentiation; CD), changes in overall wing shape (WS), and other wing defects including incomplete unfolding of the wing surfaces (WF), appearance of necrotic patches and wing cuticle with abnormal appearance or lack of rigidity (wing differentiation; WD). All cases in which the wing fails to form were described as “nW” (no wing), phenotypes affecting the bristles of the wing margin as “Q−” and phenotypes of ectopic bristle formation as “Q+.” A list of all abbreviations can be found in Figure 1, Supplementary Table S2, and in the accompanying manuscript (López-Varea *et al.* 2021). Some mutant phenotypes are more informative than others with regard to the function of the corresponding gene during wing development. In general, the phenotypes caused by the knockdown of genes with unknown developmental roles can be interpreted by comparison to those caused by perturbations in genes with known developmental roles. For example, we expect that knockdown of housekeeping genes involved in protein synthesis, modifications, trafficking, or degradation, in RNA processing or in general metabolic pathways would reduce cell viability, and when this occurs throughout imaginal development might lead to cell lethality and to a failure to form a wing. In contrast, we expect that genes involved in the modulation of the activity of signaling pathways would have phenotypes similar to perturbations in the main components of these pathways (Molnar *et al.* 2011). In any case, a mutant phenotype offers an entry point to direct further genetic and developmental characterization of the corresponding gene. In this accompanying manuscript, we present and analyzed most of the 16 molecular classes into which we classify all the *Drosophila* genes. For each class, we also defined several groups based on the molecular functions assigned for each gene and searched for phenotypic enrichment within each group.

## Materials and methods

### Wing phenotypes

We arranged the wing phenotypes of the *Gal4/UAS-RNAi* combinations presented in the preceding manuscript (López-Varea *et al.* 2021) into sets, each including the genes belonging to each molecular group. Each mutant wing was described using a set of terms that summarize its main phenotypic characteristics. The abbreviations we used are listed in Supplementary Table S2. All Gal4*/*UAS combinations were made at 25°C unless otherwise stated, and we used the Gal4 lines *sal^EPv^-Gal4* (Cruz *et al.* 2009), *nub-Gal4* or *sd-Gal4* (Calleja *et al.* 1996), and UAS-RNAi lines obtained from the Vienna *Drosophila* Resource Center (VDRC), the National Institute of Genetics Fly Stock (NIG-Fly), and the Drosophila Bloomington stock center (BDSC). The grouping and phenotypic description for the entire set of *Drosophila* genes are presented in Supplementary Table S1. A list with all wing pictures included in this manuscript is presented in Supplementary Table S3. Pictures of wings included in López-Varea *et al.* (2021) and Ostalé *et al.* (2021) are also included in Supplementary Table S3. All wing pictures we have were submitted to the Figshare repository: https://doi.org/10.6084/m9.figshare.16624645.v1; https://doi.org/10.6084/m9.figshare.16624630.v1; https://doi.org/10.6084/m9.figshare.16624603.v1; and https://doi.org/10.6084/m9.figshare.16624591.v1

### Molecular classification

In this manuscript, we described in more detail the following molecular classes defined in López-Varea *et al.* (2021): Ribosomal biology (RIB), Cell adhesion genes (CA), Protein biology (PRO), RNA biology (RNA), DNA biology (DNA), Cell signaling (SIG), Cellular metabolism (MET), Cuticle proteins (CUT), Coding genes without conserved sequence domains (CG), and Coding genes with conserved sequence domains CGh. The classes Immune responses (IMM) and Cell death (CD) are only included in Supplementary Table S1 and were not further analyzed because these genes rarely affect wing formation. Conversely, the classes Cytoskeleton (CYT), Transport across membranes (TRA), Protein secretion (PTR), and Cell division (DIV) include many genes required for wing formation and will be described elsewhere. In addition, we further classified the genes of each functional class into 3–15 groups reflecting the most prevalent aspect of each gene (Table 1). For this classification, we used GO identifiers and IP domains as well as the Flybase gene group classification and gene summaries (Thurmond *et al.* 2019). In Table 1, we present a list of all the groups and their abbreviations.

**Table 1:** Molecular classes and groups

**Ribosome (RIB)**				

**RIB-CLRP**	**RIB-CSRP**	**RIB-MLRP**	**RIB-MSRP**	**RIB-RAP**

Cytoplasmic large ribosomal subunits	Cytoplasmic small ribosomal subunits	Mitochondrial large ribosomal subunits	Mitochondrial ribosomal subunits	Ribosomal-associated proteins

**Cell signaling (SIG)**				

**SIG-BMP/TGF**	**SIG-HH**	**SIG-HSW**	**SIG-INR**	**SIG-RTK**
BMP and TGFb	Hedgehog signaling	Hippo signaling	Insulin receptor and TOR	Receptor tyrosine kinase
**SIG-JAK**	**SIG-JNK**	**SIG-NOTCH**	**SIG-TOLL**	**SIG-WNT**
Jak-Stat signaling	Jun Kinase signaling	Notch signaling	Toll signaling	Wingless signaling
**SIG-GPCR**	**SIG-NP**	**SIG-OBP**	**SIG-GR**	**SIG-OTH**
GPCR signaling	Neural Peptides	Odorant-binding proteins	Gustatory receptors	Other signaling pathways
**SIG-EC**	**SIG-IPS**	**SIG-JH**		
Ecdysone receptor	Inositol phosphate	Juvenile hormone		

**Transport across membranes (TRA)**				

**TRA-ABC**	**TRA-ICC**	**TRA-IPT**	**TRA-LGIC**	**TRA-NPC**
ATP-binding cassette transporter	Ion channels by conductane	Importins and exportins	Ligand-gated ion channels	Nuclear pore complex
**TRA-OTH**	**TRA-ATPASE**	**TRA-SLC**	**TRA-TSP**	**VHA**
Other transporters	H-ATPase	Solute carriers	Tetraspanins	Vacuolar ATPASE

**Cell adhesion (CA)**				

**CA-AJ/SJ**	**CA-CAM**	**CA-ECM**	**CA-IG**	
Adherens and septate junctions	Cell adhesion molecules	Extracellular matrix	Immunoglobulin proteins	

**Cuticular proteins (CUT)**				

**CUT-CBDCP**	**CUT-CO/VM**	**CUT-CPF**	**CUT-MU/Y**	
Chitin binding domain	Chorion and vitelline membrane	Cuticular protein families	Mucins and yellow proteins	

**Metabolism (MET)**				

**MET-AA/NT/O**	**MET-ATG**	**MET-CTR**	**MET-DTOX**	
Amino acids, nucleotides and related molecules	Autophagy	Central metabolism	Detoxification	
**MET-GLY**	**MET-LIP**	**MET-MTA**	**MET-MIT**	
Carbohydrates and glycobiology	Lipid metabolism	Metal metabolism	Mitochondrial biology	
**MET-OC**	**MET-RDX**	**MET-XEN**		
One-carbon	Oxidation-reduction	Xenobiotic		

**Protein biology (PRO)**				

**PRO-CHAP**	**PRO-GLU**	**PRO-KIN**	**PRO-PHO**	
Chaperons	Sugar modifications	Kinases	Phosphatases	
**PRO-PEP**	**PRO-PTS**	**PRO-MOD**	**PRO-UBIT**	
Proteases	Proteosome	Protein modifications	Ubiquitin ligases and transferases	

**RNA biology (RNA)**				

**RNA-MOD**	**RNA-OTH**	**RNA-BND**	**RNA-ENZ**	
mRNA modifications	Other RNA molecules	RNA-binding proteins	RNA enzymes	
**RNA-rRNA**	**RNA-SP**	**RNA-TNLF**	**RNA-tRNA**	
Ribosomal RNA	mRNA splicing	Translation initiation, elongation, and termination	Transfer RNA	

**DNA biology (DNA)**				

**DNA-CHRO**	**DNA-CMC**	**DNA-ENZ**		
Chromosomal structure proteins	Chromatin modifying complexes	DNA enzymes		
**DNA-REP**	**DNA-GTF**	**DNA-TF**		
DNA replication	General transcription factors	Sequence-specific transcription factors		

**Cytoskeleton (CYT)**				

**CYT-TUB**	**CYT-MYO**	**CYT-ACT**		
Tubulin	Myosin	Actin		

**Protein transport across membranes (PTR)**				

**PTR-EN**	**PTR-ER**	**PTR-GA**		
Endocitosis	Endoplasmic reticulum	Golgi apparatus		
**PTR-GPI/SRP**	**PTR-LY/PE**	**PTR-VT**		
GPI anchor and signal recognition particle	Lysosome and Peroxisome	Vesicle-mediated transport		

**Cell division (DIV)**				

**DIV-CC**	**DIV-MIT**	**DIV-MEI**		
Cell cycle	Mitosis	Meiosis		
CG	CG(h)	CD	IMM	
**Gene with no homology**	**Gene with InterPro domain**	**Cell death**	**Immunology**	

Name, abbreviation, and number of genes included in each functional/molecular class and name, abbreviation, and number of genes of each functional group included in each class.

### Gene expression analyses and *in situ* hybridization data

To define whether a gene is or not expressed in the wing disc, we used RNA-seq data (Flegel *et al.* 2016; reads from run SRR3478156) and microarray data (GeneChip™ *Drosophila* Genome 2.0 Array; Affymetrix) obtained from mRNA extracted from dissected third instar wing imaginal discs (Organista *et al.* 2015). The *in situ* hybridizations pictures correspond to a collection of 635 experiments published in Hevia *et al.* (2017), Molnar *et al.* (2012), and Organista *et al.* (2015). All the *in situ* hybridization experiments were carried out in our laboratory. The expression patterns were classified as no expression (NE), ubiquitous expression (GEN), and restricted expression (PAT). Any expression pattern was defined as “ubiquitous” when the staining occurs throughout the wing disc at similar levels that were consistently higher than those observed in *in situ* experiments carried out with *sense* probes. A list with all wing imaginal disc *in situ* hybridization pictures included in this manuscript is presented in Supplementary Table S3.

## Results and discussion

We screened in the fly wing UAS-RNAi lines targeting 10,920 *Drosophila* genes of which 7036 were estimated as being expressed in the wing disc. We also classified all *Drosophila* genes into 16 functional classes encompassing the more functionally relevant aspect of each gene. For this classification, we relied on available GO annotations, IP domains, and bibliographical searches. When a gene could not be classified unambiguously into only one functional class, we selected one based on prior knowledge about the gene function during imaginal development. In this accompanying manuscript, we present a detailed description of 10 of these functional classes, aiming to identify whether they have preferential phenotypic characteristics. We also define within each of these classes several groups based in the known characteristics of each gene (Table 1). This classification was based when possible on the “gene group” annotation from Flybase (Thurmond *et al.* 2019).

### Ribosomal genes

We include in this class all genes encoding components of the cytoplasmic and mitochondrial large and small ribosomal proteins (54 genes for RIB-CLRP, 39 for RIB-CSRP, 47 for RIB-MLRP, and 28 for RIB-MSRP; structural components of ribosomes; see Supplementary Table S1.1). We also included in this class, a group of 67 genes annotated as ribosomal binding and assembly proteins, ribosomal biogenesis, and other genes encoding protein domains suggestive of a role in ribosome biology (Ribosomal-associated proteins; RIB-RAP, Figure 1A;Supplementary Table S1.1). Most of Ribosomal genes are expressed in the wing disc (95%), and this expression occurs at high levels (Figure 1B;Supplementary Table S1.1). In those cases for which we have *in situ* hybridization data, the expression was robust and ubiquitous in the wing disc and other tissues (Figure 1, D–G). Mutations in ribosomal genes are lethal in homozygosis and they cause dominant *Minute* phenotypes consisting in the formation of bristles of smaller size and developmental delay (Marygold *et al.* 2007). The knockdown phenotypes of ribosomal genes (*UAS-Dicer2/+; nub-Gal4/UAS-RNAi* combinations) mostly consist in late larval or early pupal lethality (68%; Figure 1, B and C). All combinations of cytoplasmic ribosomal genes resulting in viable adults developed rudimentary wings or a total absence of the wing (Figure 1, C and H–L). When the knockdown was restricted to the central region of the wing (*UAS-Dicer2/+; sal^EPv^-Gal4/UAS-RNAi* combinations) this region generally failed to form (Supplementary Table S1; Figure 1K’), although we found some cases in which the wing was fully formed but its size was reduced (Figure 1, H–H’). In summary, genes encoding components of the ribosome are, as expected, expressed at high levels and ubiquitously in the wing disc, and the most frequent phenotypes resulting from Ribosomal genes knockdowns (larval and pupal lethality and loss of wing tissue) are compatible with their requirements for epithelial cell viability.

### Cell adhesion genes

**Figure 2:**
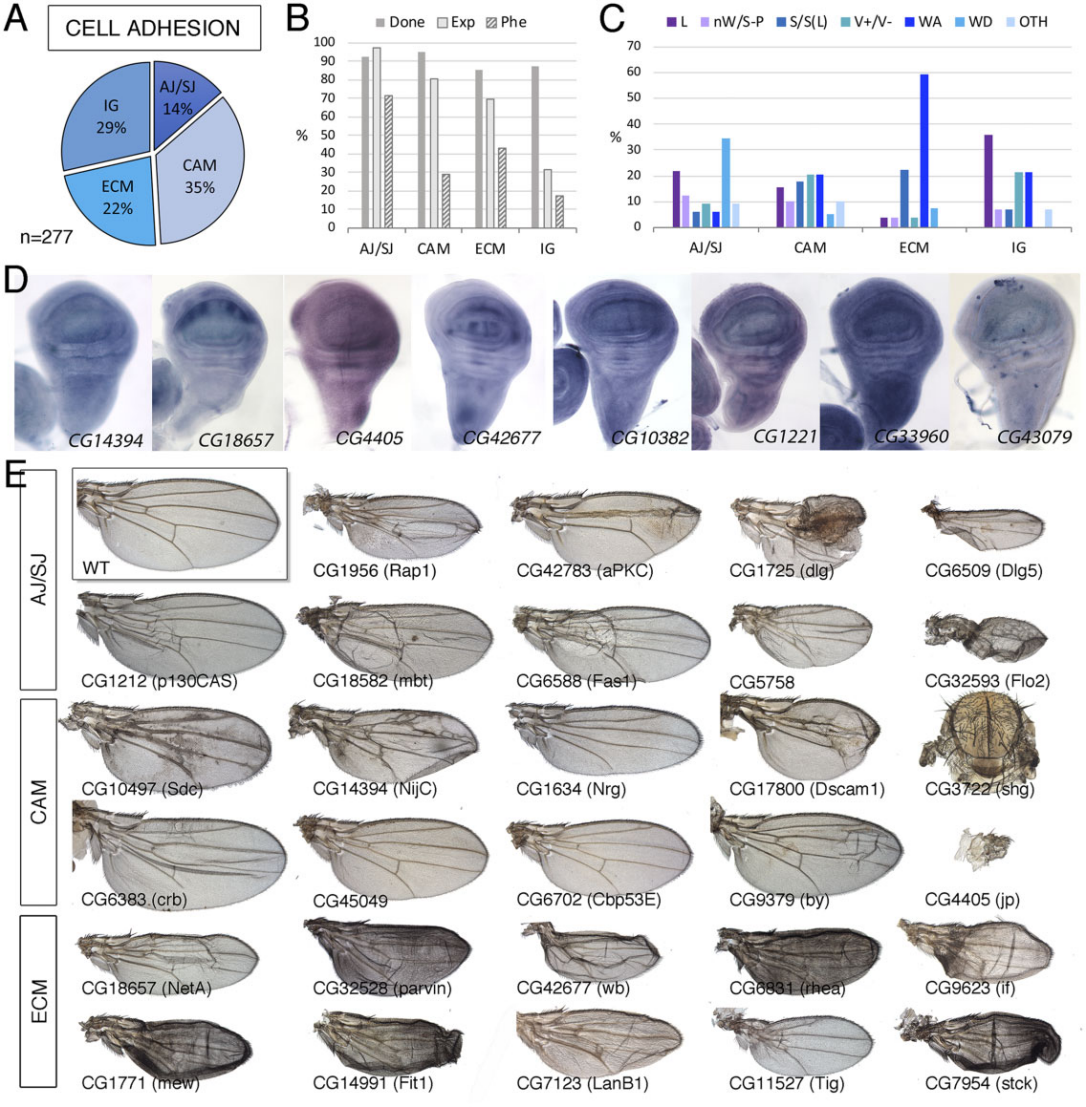
Cell adhesion class. (A) Distribution of the 277 genes included in the Cell adhesion class into the groups Adherent and septate junctions (AJ/SJ), Cell adhesion molecules excluding Immunoglobulin domain-containing proteins (CAM), Extracellular matrix adhesion (ECM), and Cell adhesion molecules containing Immunoglobulin domains (IG). (B) Percentages of genes for which we tested its knockdown phenotype (Done; dark gray column), of genes expressed in the wing disc (Exp; light gray column), and of genes with a lethal or visible phenotype in knockdown conditions (Phe; striped column) for the classes AJ/SJ, CA, ECM, and IG. (C) Frequency of lethal (L) and visible mutant phenotypes observed in the AJ/SJ, CA, ECM, and IG groups. Notice the high frequency of WA phenotypes in the ECM group compared to other groups. (D) Examples of expression patterns in the wing disc for different members of the CA class (name of each gene in the bottom right corner of each picture). The genes CG18657 and CG42677 show restricted expression to the ventral compartment and to the interveins, respectively. *In situ* hybridizations pictures correspond to experiments published as supplementary material in Molnar *et al.* (2012), Organista *et al.* (2015), and Hevia *et al.* (2017). (E) Wing phenotypes of representative examples of knockdowns of CA genes belonging to the AJ/SJ, CA, and ECM groups. All genotypes are from *UAS-Dicer2/+; nub-Gal4/UAS-RNAi* flies. The name of each gene is given in the bottom-left corner of each wing picture.

The regulation of cell-to-cell adhesion is critical for the generation of tissue shape and the maintenance of epithelial integrity, and in the imaginal epithelium plays key roles during signaling, cell division, apoptosis, and pupal differentiation (Gumbiner 1996; Bökel and Brown 2002). We included in CA all genes encoding cytoplasmic or membrane-associated proteins related to cell-to-cell adhesion and to cell to extracellular matrix adhesion. Based on their structural and/or functional characteristics, we further defined four large groups: adherens/septate junctions (CA-AJ/SJ; 38 genes; Figure 2A), extracellular matrix (CA-ECM; 62 genes; Figure 2A), proteins containing immunoglobulin domains (CA-IG; 79 genes; Figure 2A), and other cell adhesion and adhesion-related molecules (CA-CAM; 98 genes; Figure 2A). The group with a higher percentage of genes required for wing formation is those related to the formation of adherens and septate junctions (71% genes; Figure 2B). These genes are also generally expressed in the wing disc (97%; Figure 2B) and the phenotype of their knockdown includes mostly larval or pupal lethality with a loss of wing tissue and defects in wing cuticle differentiation (Figure 2, C and E; Supplementary Table S1.2). Genes encoding proteins related to the formation of the cell matrix or proteins required for interactions with the cell matrix are also expressed in the wing disc (69%; Figure 2B) and the phenotype more often found after knockdown consisted in the formation of blisters in the wing cuticle (“WA”; Figure 2, C and E; Supplementary Table S1.2). These blisters results from failures in the adhesion of the dorsal and ventral wing surfaces during pupal development, once the two wing surfaces are confronted to each other through their basal cell membranes (Fristrom *et al.* 1993; Bilousov *et al.* 2014). The blister could affect the entire wing, giving the wing the appearance of an inflated balloon, or only a fraction of the wing, resulting in the formation of blisters with variable extension. The region most frequently affected is centered along the posterior cross vein (Figure 2E). In general, these blistery wings appeared with variable frequency depending on the particular gene knockdown.

The last groups of genes we included in the cell adhesion class encode cell adhesion molecules of the cadherin (Zartman *et al.* 2009), Fasciclin, Dscam, and Immunoglobulin families (Vogel *et al.* 2003), as well as a variety of other proteins with a GO description indicative of a role in cell adhesion (Supplementary Table S1.2). These genes were included in two groups: cell adhesion molecules with immunoglobulin domains (CA-IG; 79 genes) and other cell adhesion molecules and proteins with predicted role in cell adhesion (CA-CAM; 98 genes). These genes are frequently not essential for wing development (CA-CAM and CA-IG groups; Figure 2B), although a large fraction of CA-CAM genes are expressed in the wing disc (81%). In contrast, only 32% of genes encoding proteins with Immunoglobulin domains are expressed in this tissue (Figure 2B). Furthermore, only 17% of these genes result in lethality or a visible phenotype (Figure 2B). The more frequent phenotypes we observed in genes of these groups (cell adhesion molecules and immunoglobulin-containing proteins) were pupal lethality, wing size defects, altered vein differentiation, and formation of wing blisters (Figure 2, C and E). The expression of cell adhesion genes occurs in a ubiquitous manner, but many cases of increased expression in the interveins or the dorsal or ventral wing surfaces are also observed (Figure 2D). In summary, we find enrichment of two phenotypes for genes belonging to the CA class: lethality in the case of CA-AJ/SJ and blisters in the case of CA-ECM, indicating requirements for cell viability and for the attachment of the dorsal and ventral wing surfaces, respectively. For the most part, the function of cell adhesion molecules containing immunoglobulin domains is dispensable during wing development.

### Protein biology

**Figure 3:**
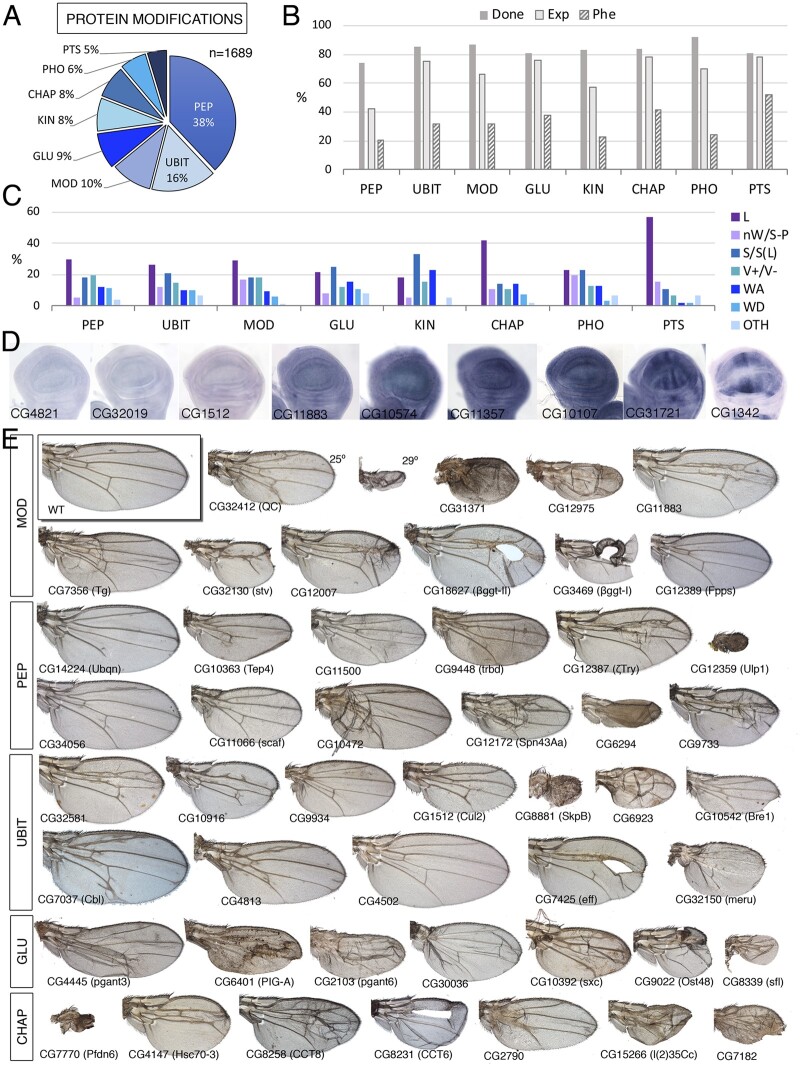
Protein class. (A) Distribution of the 1689 genes included in the Protein class into the groups peptidases (PEP), Ubiquitin ligases and transferases (UBIT), post-transcriptional protein modifications other than sugar, kinases, and phosphatases and ubiquitinylases (MOD), protein modifications by sugars (GLU), kinases (KIN), chaperons (CHAP), phosphatases (PHO), and components of the proteasome (PTS). (B) Percentages of genes for which we tested its knockdown phenotype (Done; dark gray column), of genes expressed in the wing disc (Exp; light gray column), and of genes with a lethal or visible phenotype in knockdown conditions (Phe; striped column) for the classes PEP, UBIT, MOD, GLU, KIN, CHAP, PHO, and PTS. (C) Frequency of lethal (L) and visible mutant phenotypes (colored columns) observed in the classes PEP, UBIT, MOD, GLU, KIN, CHAP, PHO, and PTS groups. Note the high frequency of lethal combinations in the CHAP and PTS classes and of wing size phenotypes in the KIN class compared to other groups. (D) Examples of expression patterns in the wing disc for different members of the Protein class (name of each gene in the bottom-left corner of each picture). The genes are ordered from not expressed in the wing disc (left) to ubiquitous and patterned expression (right). *In situ* hybridizations pictures correspond to experiments published as supplementary material in Molnar *et al.* (2012), Organista *et al.* (2015), and Hevia *et al.* (2017). (E) Representative wings of knockdowns for genes of the MOD (up), PEP, UBIT, GLU, and CHAP groups belonging to the Protein class. All genotypes are from *UAS-Dicer2/+; nub-Gal4/UAS-RNAi* flies. The gene names are displayed in the bottom-left corner of each wing picture.

**Figure 4:**
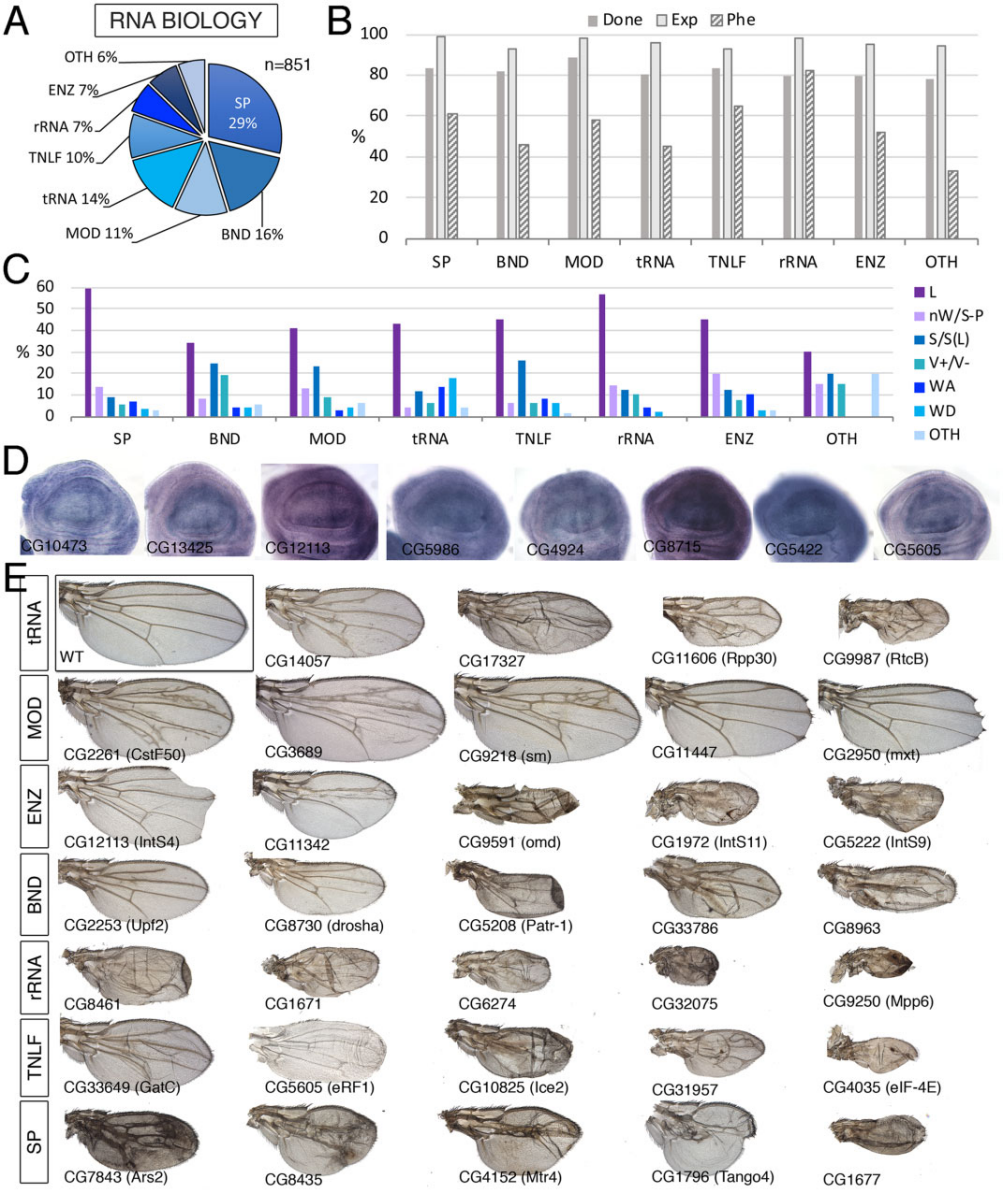
RNA class. (A) Distribution of the 851 genes included in the RNA class into the groups “Splicing” (SP), RNA binding (BND), mRNA processing (MOD), tRNA (tRNA), Translation initiation factors (TNLF), ribosomal RNA (rRNA), RNA enzymes (ENZ), and RNA molecules distinct to tRNA, mRNA, and rRNA (OTH). (B) Percentages of genes for which we tested its knockdown phenotype (Done; dark gray column), of genes expressed in the wing disc (Exp; light gray column), and of genes with a lethal or visible phenotype in knockdown conditions (Phe; striped column) for the groups SP, BND, MOD, tRNA, TNLF, rRNA, ENZ, and OTH. (C) Frequency of lethal (L) and visible mutant phenotypes observed for the groups SP, BND, MOD, tRNA, TNLF, rRNA, ENZ, and OTH. Note the high frequency of lethal combinations in the SP, tRNA, TNLF, ENZ, and rRNA groups and of wing size phenotypes in the BND, MOD, and TNLF groups compared to other groups. (D) Examples of expression patterns in the wing disc for different members of the RNA class (name of each gene in the bottom-left corner of each picture). Most genes are expressed in a ubiquitous manner in the wing disc. *In situ* hybridizations pictures correspond to experiments published as supplementary material in Molnar *et al.* (2012), Organista *et al.* (2015), and Hevia *et al.* (2017). (E) Representative wings of knockdowns of RNA genes belonging, from top to bottom, to the tRNA (up), MOD, ENZ, BND, rRNA, TNLF, and SP (bottom) groups. Genotypes for CG3689, CG9218, CG11342, CG8435, CG4152, and CG1796 genes are from *UAS-Dicer2/+; sal^EPv^-Gal4/UAS-RNAi* flies. The remaining are from *UAS-Dicer2/+; nub-Gal4/UAS-RNAi* flies. The name of each particular gene is given in the bottom-left corner of each wing picture.

We include in this class a large number of genes (1689) encoding enzymes which activity results in post-translational modifications to other proteins (Supplementary Table S1.3). Most of these proteins are poorly characterized, and, because they could affect multiple targets, they contribute to a variety of cellular processes. We have considered as members of the Protein biology class the following groups: Peptidases (PRO-PEP; 642 genes; Figure 3A), Ubiquitin ligases and transferases (PRO-UBIT; 270 genes; Figure 4A), Chaperones involved in protein folding (PRO-CHAP; 134 genes; Figure 3A), proteins involved in glucids modifications (PRO-GLU; 148 genes; Figure 3A), Kinases and Phosphatases that could not be easily ascribed to other functional classes based on their function (PRO-KIN and PRO-PHO; 137 and 109 genes, respectively; Figure 3A), genes encoding proteins involved in other protein modifications such as palmitoylation, geranylation, neddylation, arginylation, polyglycylation, sumoylation, and lipoylation, among others (PRO-MOD; 170 genes; Figure 3A) and components of the proteasome (PRO-PTS; 79 genes; Figure 3A). The Kinase and phosphatase groups are treated separately in the accompanying manuscript (Ostalé *et al.* 2021). All other groups within the protein biology class behave in a similar manner. Thus, a significant fraction of these genes (60%) are expressed in the wing disc, ranging from 42% (Protein Peptidases; Figure 3B) to 79% (components of the proteasome; Figure 3B). The fraction of genes whose knockdown in the wing disc results in lethality or in a mutant phenotype is low (29% for all members of the protein biology class; shown by group in Figure 3B), and is only higher than the average for this class or for the genome in the case of components of the proteasome and protein folding groups (57% and 41%; Supplementary Table S1.3, Figure 3, B and C, and Supplementary Figure S1B). The fraction of genes with a mutant phenotype in knockdown conditions is particularly low in the case of proteases (21%; Figure 3B) and protein kinases and phosphatases (23% and 24%, respectively), possibly due to gene redundancy or because these proteins are involved in biological processes that are not critical for imaginal development. There are no clear enrichments in specific mutant phenotypes for any group within the class (Figure 3, C and E). The main exceptions are lethality, which is more frequent in the proteasome and chaperon groups (57% and 42%; Figure 3, C and E and Supplementary Figure S1B), and wing adhesion phenotypes, which are more frequent in the kinase group compared to the other groups (Figure 3, C and E and Supplementary Figure S1B). The expression of these genes visualized by *in situ* hybridization includes not only multiple examples of ubiquitous expression (Figure 3D) but also cases of restricted expression related to the development of vein and intervein territories (Figure 3D). In summary, only genes encoding components of the proteasome and genes encoding chaperons show a general requirement for wing development, resulting in loss-of-function conditions in phenotypes compatible with reduced cell viability.

### RNA Biology

We classified 851 genes as being fundamentally related to the synthesis, processing, and modification of RNA molecules (Figure 4A;Supplementary Table S1.4). We further divided this class into eight groups: genes involved in the synthesis and maturation of ribosomal RNA (RNA-rRNA, 58 genes), genes related to the synthesis and modification of tRNAs (RNA-tRNA; 118 genes), general translation initiation, elongation, and termination factors (RNA-TNLF, 83 genes), genes encoding members of the mRNA splicing machinery (RNA-SP; 244 genes), genes encoding enzymes catalyzing mRNA modifications (RNA-MOD; 99 genes), RNA enzymes (RNA-ENZ; 58 genes), genes encoding RNA-binding proteins (RNA-BND; 141 genes), and genes related to the biology of other RNA molecules such as piRNA, snRNA, siRNA, and snoRNA (RNA-OTH, 50 genes).

**Figure 5:**
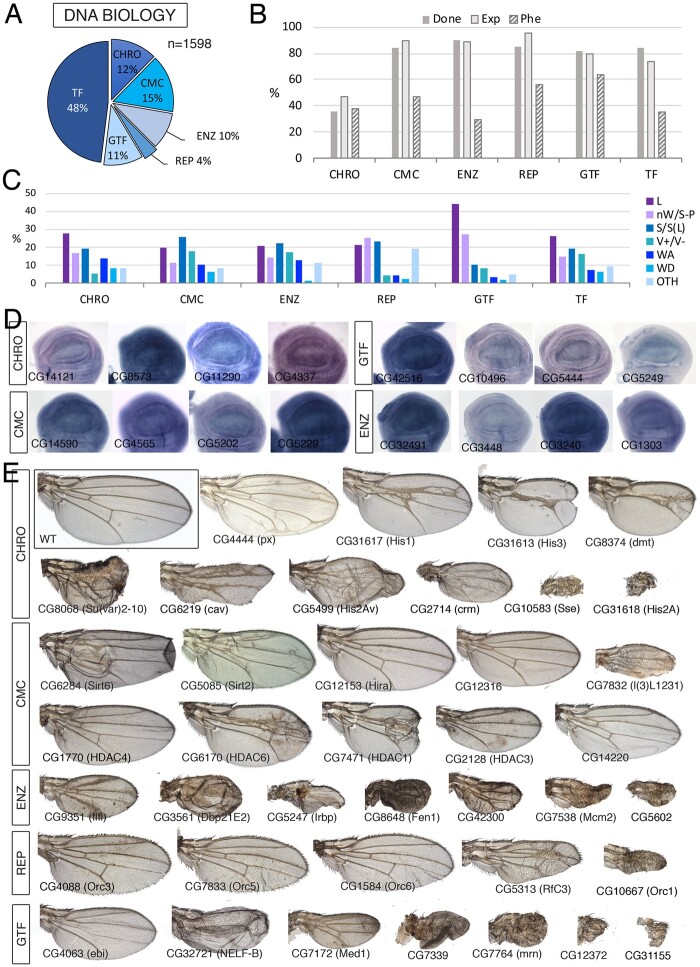
DNA class. (A) Distribution of the 1598 genes included in the DNA class into the groups “Chromosomal maintenance and structure” (CHRO), Chromatin modifying proteins and protein complexes (CMC), DNA modifying enzymes (ENZ), DNA replication (REP), General transcription factors (GTF), and sequence-specific transcription factors (TF). (B) Percentages of genes within each class for which we tested its knockdown phenotype (Done; dark gray column), of genes expressed in the wing disc (Exp; light gray column), and of genes with a lethal or visible phenotype in knockdown conditions (Phe; striped column). (C) Frequency of lethal (L) and visible mutant phenotypes observed in the classes CHRO, CMC, ENZ, REP, GTF, and TF. Notice the high frequency of lethal combinations in the GFT class, of wing veins effects in the CMC and ENZ classes. The increase in other phenotypes (OTH) observed in the REP class corresponds to cell differentiation phenotypes. (D) Examples of expression patterns in the wing disc for different members of the DNA class (name of each gene in the bottom-left corner of each picture). Most genes are expressed in a ubiquitous manner in the wing disc. *In situ* hybridizations pictures correspond to experiments published as supplementary material in Molnar *et al.* (2012), Organista *et al.* (2015), and Hevia *et al.* (2017). (E) Representative wings of knockdowns of DNA genes belonging, from top to bottom, to the CHRO (up), CMC, ENZ, REP, and GTF (bottom) classes. All genotypes except *His1:CG31617*, *Cap-G*, *His3:CG31613*, and *dmt* are from *UAS-Dicer2/+; nub-Gal4/UAS-RNAi* flies. *His1:CG31617*, *Cap-G*, *His3:CG31613*, and *dmt* are from *UAS-Dicer2/+; sal^EPv^-Gal4/UAS-RNAi* flies. The name of each gene is given in the bottom-left corner of each wing picture.

The RNA class shares several characteristics with the Ribosome class. Thus, RNA genes are generally expressed in the wing disc (96%; Figure 4B), and they are mostly expressed at high levels and in a ubiquitous manner (Figure 4D). They also include a large fraction of genes required for imaginal development (56% aggregate; shown by group in Figure 5B). The fraction of genes with a lethal or mutant phenotype is maximal for RNA-rRNA encoding genes (83%) and minimal for genes classified as RNA-OTH (33%). The most frequently found phenotypes were lethality (48%) and wing size defects (16%) (Figure 4, C and E). The frequency of other phenotypes was low in all groups (Figure 4C), although the RNA-tRNA class shows enrichment in the WA and WD phenotypes (Supplementary Figure S1B). In summary, loss-of-function conditions of genes included in the RNA class, mostly in the splicing, translation, and ribosomal RNA groups (RNA-SP, RNA-TNLF, and RNA-rRNA) share a large frequency of phenotypes related to reduced cell viability (larval and pupal lethality, loss of wing tissue and strong defects in wing size and pattern) and reduced cell proliferation (reduced wing size). As expected these genes are mostly expressed ubiquitously and at high levels in the wing disc epithelium.

### DNA biology

We classified 1598 genes as being involved in DNA biology (Figure 5A;Supplementary Table S1.5). This class was further divided into six large groups including sequence-specific transcription factors (DNA-TF, 768 genes), general transcription factors (DNA-GTF, 168 genes), DNA replication (DNA-REP, 62 genes), Chromatin modifying complexes (DNA-CMC, 246 genes), enzymes affecting DNA conformation and maintenance (DNA-ENZ, 157 genes), and chromosomal structure and maintenance proteins (DNA-CHRO, 197 genes). For all these groups, we find similar frequencies of expressed genes (average 76%; Figure 5B) and similar frequency of knockdowns with lethality or a mutant phenotype (average 41%; Figure 5B). The only exception is the chromosomal genes class, which has much lower values of expressed genes (47%) and genes with a mutant phenotype (18%, Figure 5B). This last group includes all histone coding genes (114 genes), which are present in multiple copies grouped in gene clusters. In general, only one (*His2A* 1/20; *His2B* 1/23; *His3* 1/23 and *His4* 1/22) or two genes (*His1* 2/23) encoding each Histone type are expressed in the wing disc. For these genes (*His1:CG31617*, *His2A:CG31618*, *His2B:CG17949*, *His3:CG31613*, and *His4:CG31611*), the knockdown results in larval (*His1:CG31617* and *His2B:CG17949*) or pupal (*His2A:CG31618*) lethality, as well as strong effects in wing size (*His1:CG31617*, *His2A:CG31618*, and *His3:CG31613*). The knockdowns of other genes encoding Histone variants (*His3.3A and His2Av*) reduce wing size (Figure 5E). When we exclude all Histone coding genes not expressed in the wing disc from the Chromosomal group, the overall expression and phenotype parameters are similar to those of other groups in the DNA class (not shown).

The larger group within the DNA class corresponds to transcription factors and proteins with sequence-specific DNA-binding domains that could contribute to the regulation of gene expression (DNA-TF; 768 genes). Transcription factors play key roles during wing imaginal development, contributing to the subdivision of the wing disc in domains of gene expression corresponding to the future anatomical regions of the wing and thorax (Ostalé *et al.* 2018). For this reason, and because of the large number of TF expressed in the disc, they will be analyzed separately (Ostale *et al.* in preparation). The most frequent phenotypes we found within the DNA class are pupal lethality, which is particularly prominent within the DNA-GTF (44%; Figure 5C), defects in wing size and pattern, including those cases in which the wing fails to form in the DNA-GTF and DNA-REP groups (27% and 26%, respectively; Figure 5, C and E), reductions of wing size with minor or no effect on vein patterning in the DNA-CMC, DNA-REP groups, and DNA-ENZ groups (26%, 23%, and 22%, respectively; Figure 5, C and E) and defects in vein differentiation in the DNA-CMC group, DNA-ENZ and DNA-TF groups (18%, 17%, and 16%, respectively; Figure 5, C and E). All other phenotypes appear with similar frequencies in all different groups, varying from 1.6% to 19% (Figure 5C). Most expression patterns observed for genes of the DNA class, excluding sequence-specific transcription factors, were ubiquitous in the wing disc (Figure 5D).

### Cell signaling

**Figure 6:**
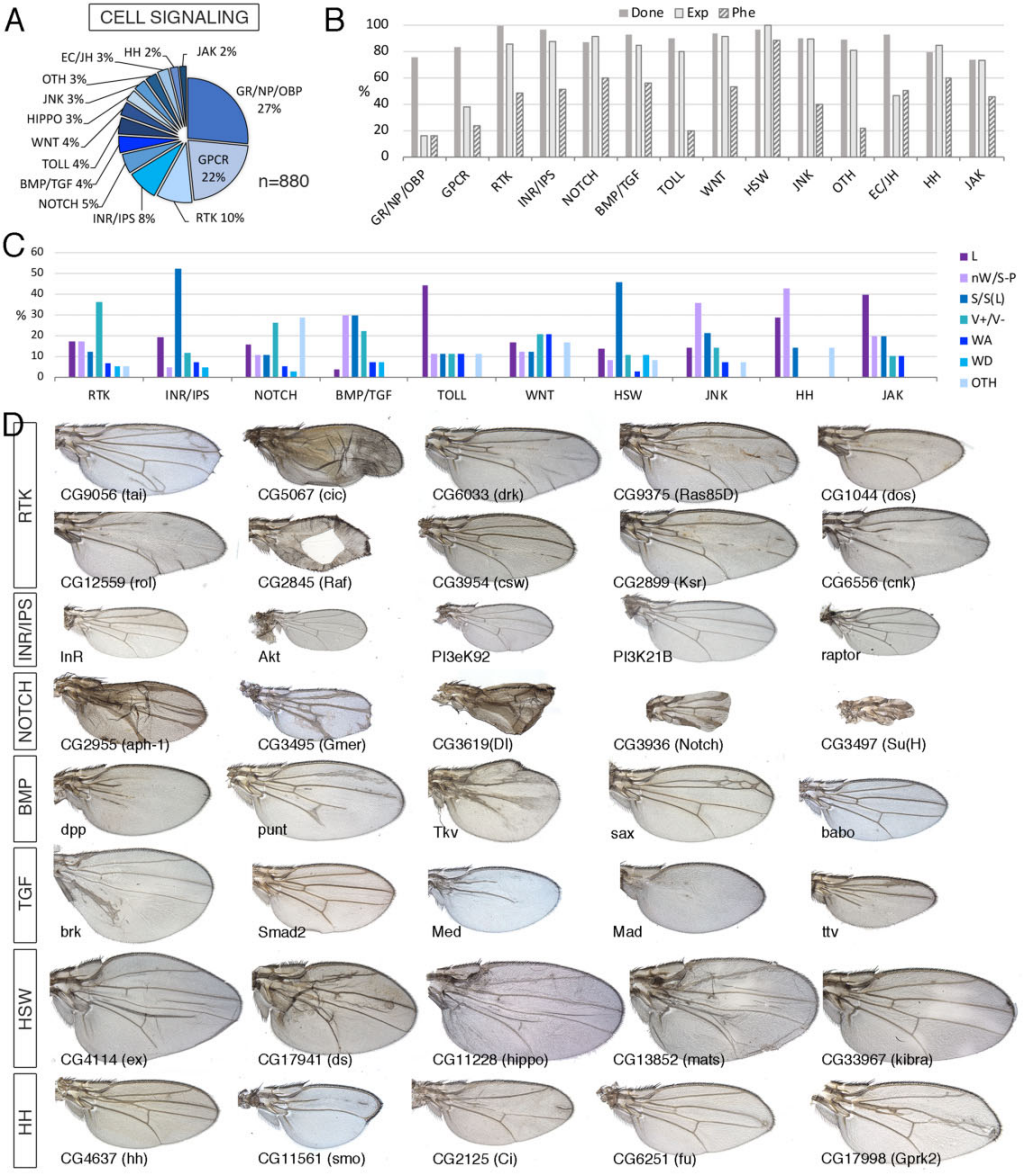
Signaling class. (A) Distribution of the 880 genes included in the Signaling class into the groups Gustatory receptors, Neural peptides, and odorant-binding proteins (GR/NP/OBP), G-Protein coupled receptors (GPCR), Receptor tyrosine kinase pathways (RTK), Insulin receptor and Inositol phosphate signaling pathways (INR), Notch signaling pathway (NOTCH), BMP and TGFβ signaling pathways (BMP/TGF), Toll receptors signaling pathway (TOLL), Wnt signaling pathway (WNT), Hippo/Salvador/Warts signaling pathways (HIPPO), Jun Kinase signaling pathway (JNK), ecdysone and Juvenile hormone signaling (EC/JH), Hedgehog signaling (HH), JAK/STAT signaling (JAK), and other signaling molecules (OTH). (B) Percentages of genes for which we tested its knockdown phenotype (Done; dark gray column), of genes expressed in the wing disc (Exp; light gray column), and of genes with a lethal or visible phenotype in knockdown conditions (Phe; striped column) for the groups shown in (A). (C) Frequency of lethal (L) and visible mutant phenotypes observed in the classes RTK, INR/IPS, NOTCH, BMP/TGF, TOLL, WNT, HSW, JNK, HH, and JAK. Note the high frequency of size defects in the INR/IPS and HIPPO classes, of vein defects in the RTK and BMP/TGF classes and of loss of wing tissue or strong defects in wing size and pattern in the HH and JNK classes. The increase in other phenotypes (OTH) observed in the NOTCH class corresponds to wing margin defects. (D) Representative wings of knockdowns Signaling class genes belonging, from top to bottom, to the RTK, INR/IPS, NOTCH, BMP, TGF, HSW, and HH classes. Wings corresponding to *drk*, *Ras85D*, *dos*, *rl*, *csw*, *ksr*, and *cnk* are from *UAS-Dicer2/+; sal^EPv^-Gal4/UAS-RNAi* flies and the rest from *UAS-Dicer2/+; nub-Gal4/UAS-RNAi* flies. The name of each particular gene is given in the bottom-left corner of each wing picture.

In this class, we included all components of the Ecdysone receptor (SIG-EC; 20 genes), Juvenile hormone (SIG-JH; 6 genes), Hedgehog (SIG-HH; 19 genes), Hippo/Salvador/Warts (SIG-HSW; 27 genes), Bone morphogenetic protein and Transforming growth factor β (SIG-BMP/TGF; 39 genes), Insulin receptor and Inositol signaling (SIG-INR/IPS; 72 genes), Jak-Stat (SIG-JAK; 15 genes), Jun Kinase (SIG-JNK; 28 genes), Notch (SIG-NOTCH; 46 genes), Toll (SIG-TOLL; 39 genes), Wingless (SIG-WNT; 32 genes), and Receptor tyrosine kinase (SIG-RTK; 84 genes) pathways (Figure 6A;Supplementary Table S1.6). We also include all Neural peptides (SIG-NP; 45 genes), Odorant-binding proteins and gustatory receptors (SIG-OBP and SIG-GR, 128 and 60 genes, respectively), and a large collection of genes encoding proteins with the typical structure of G-protein coupled receptors (SIG-GPCR; 193 genes). A small number of genes (26) encoding molecules related to TNF, Robo, Nitric oxide, Semaphorin, NFAT, and Ephrin signaling were classified as “SIG-OTH” (Supplementary Table S1.6; Figure 6A).

Annotated members of the canonical signaling pathways are generally expressed in the wing disc, ranging from 80% for components of the Toll signaling pathway to 100% for members of the Hippo pathway (Figure 6B). All these pathways have been thoroughly characterized in their functions during wing imaginal development in the regulation of wing growth, vein patterning and differentiation, and dorsoventral boundary formation (Molnar *et al.* 2011). As these signaling pathways are modules formed by proteins linked by protein–protein interactions resulting in unique outputs, it is expected that knockdown of any component of the pathway results in similar phenotypes varying only in their strength. For example, the knockdown of most members of the BMP and TGFβ pathways results in wings reduced in size and accompanied with loss and extra-vein phenotypes in the case of BMP components (Figure 6, C and D). These phenotypes reveal the known functions of these pathways in promoting wing growth (BMP and TGF), vein patterning (BMP), and vein differentiation (BMP) (de Celis 1997; Brummel *et al.* 1999). The function of the Hh signaling pathway is required to regulate the expression of the BMP ligand *dpp* and other genes in a central region of the wing disc abutting the anteroposterior compartment boundary (Tabata and Kornberg 1994; Zecca *et al.* 1995). As expected, knockdown of Hh components results in the formation of smaller than normal wings that lose some structures located between the veins L3 and L4 (Figure 6D).

Members of the Notch pathway also share a similar set of phenotypes, consisting in vein thickening, loss of wing margin structures, and reduced wing size (Figure 6, C and D and Supplementary Figure S1B). All these phenotypes are reminiscent of *Notch* loss-of-function alleles (de Celis and García-Bellido 1994). Knockdown of components of the Epidermal growth factor receptor pathway (RTK) results as expected in two opposite phenotypes regarding vein formation. Thus, the knockdowns of genes promoting ERK phosphorylation (*EGFR*, *drk*, *Ras85D*, *dos*, *rol*, *Raf*, *csw*, *ksr*, and *cnk*) cause loss of veins and reduced wing size, and in strong conditions lead to the total absence of the wing (Figure 6D). These phenotypes are compatible with the known function of EGFR signaling promoting vein differentiation and wing growth (Sturtevant *et al.* 1993; Simcox 1997). On the other hand, knockdown of pathway members that antagonize ERK phosphorylation [*tai and MKP3*; see Molnar and de Celis (2013) and Ruiz-Gómez *et al.* (2005), respectively] or that repress gene expression in the absence of pathway activation (*cic* see Jiménez *et al.* 2012), result in the formation of ectopic veins (Figure 6D). Knockdown of different members of the InR signaling pathway affect cell size and wing size, without any effect on the pattern of veins (Figure 6, C and D). These phenotypes are compatible with the known function of InR signaling to promote cell growth in imaginal cells (Edgar 2006). The Hippo pathway is required to promote cell proliferation in all imaginal discs (Halder and Johnson 2011). Most components of the pathway act to promote the phosphorylation of the transcriptional coactivator Yorkie, causing its inactivation by sequestering this protein in the cytoplasm (Halder and Johnson 2011). Accordingly, knockdown of Yorkie upstream components results in the formation of larger and rounder wings missing the cross veins (Figure 6D). The expression of most member of this pathway occurs at high levels in the wing disc (Figure 6B).

Other genes we classified as members of the signaling class were GPCR (193 genes) and gustatory receptors, neural peptides, and odorant-binding proteins (233 genes). This is a very numerous group (426 genes; Supplementary Table S1.6), but only a small fraction of these genes are expressed in the wing disc (37% for SIG-GPCR and 16% for SIG-GR, SIG-NP, and SIG-OBP; Figure 6B). The number of knockdowns resulting in lethality or a mutant phenotype was significantly lower than the average for the signaling class (15% and 23% *vs* 36%). We also noticed that the majority (14/32 for SIG-GPCR, 10/10 for SIG-GR, 1/2 SIG-NP, and 13/15 for SIG-OBP) of knockdowns resulting in a mutant phenotype correspond to genes that were classified as not expressed in the wing disc, suggesting that these phenotypes may correspond to off-target effects. In contrast, the number of possible off-target effects for members of the canonical signaling pathways was very low (14/176). In summary, we find a set of coherent phenotypes for the different components of each signaling pathway. The phenotypes for components of each pathway vary mostly in strength and are very much compatible with the known requirements of these pathways during wing development. Perhaps not surprisingly, the majority of GPCR receptors, neural peptides, and odorant-binding proteins are not required for wing development.

### Metabolism

**Figure 7:**
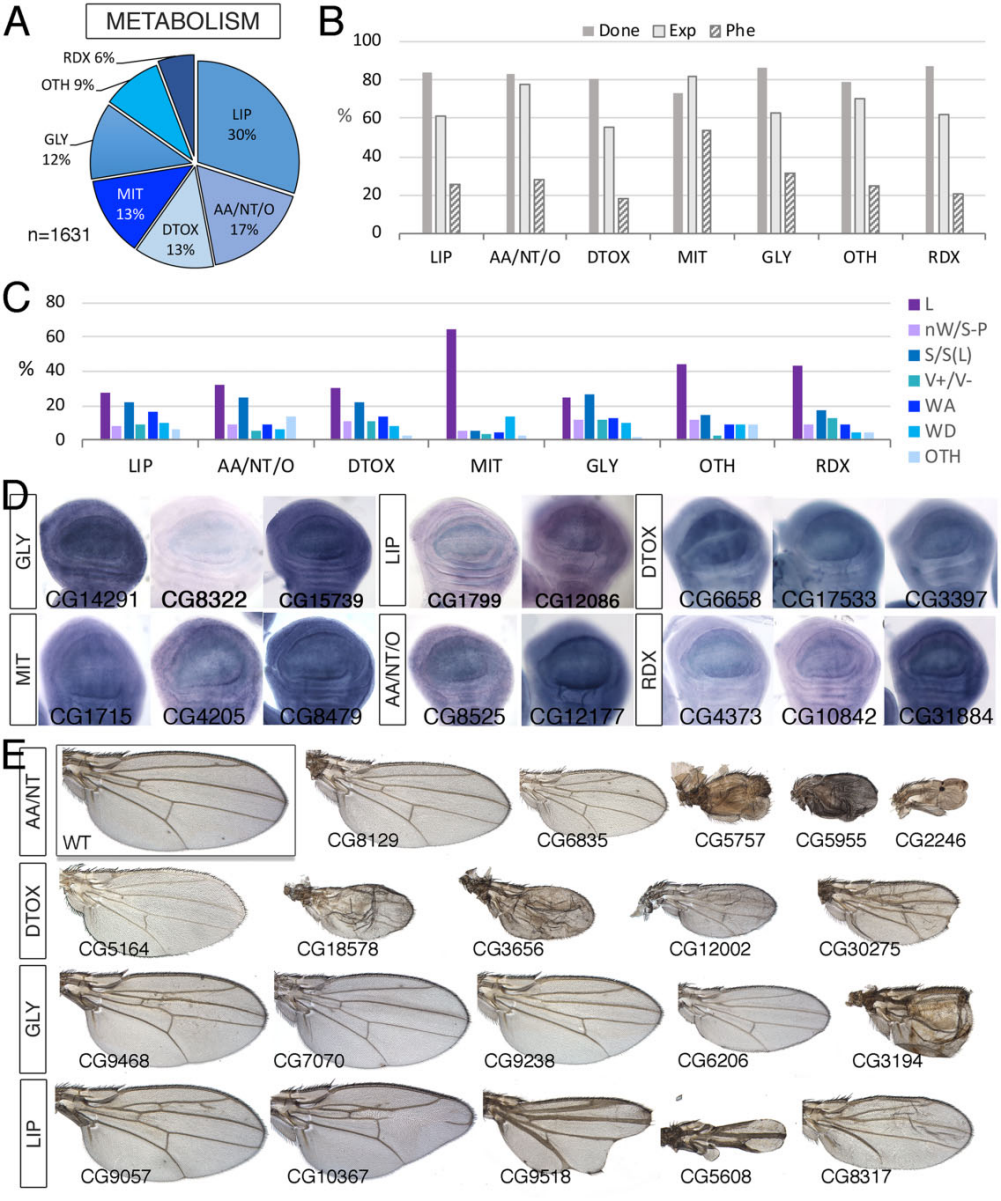
Metabolism class. (A) Distribution of the 1631 genes included in the Metabolism class into the groups Lipid metabolism (LIP), amino acid, nucleotides, and other small molecules metabolism (AA/NT/O), Detoxifying enzymes (DTOX), Mitochondrial biology and mitochondrial respiratory chain (MIT), Sugars metabolism (GLY), Oxido-reduction metabolism (RDX), and other metabolic processes including Central metabolism, autophagy, one-carbon metabolism, metal metabolism, and xenobiotic metabolism (OTH). (B) Percentages of genes within each group for which we tested the knockdown phenotype (Done; gray column), of genes expressed in the wing disc (Exp; light gray column), and of genes with a lethal or visible phenotype in knockdown conditions (Phe; striped column). (C) Frequency of lethal (L) and visible mutant phenotypes observed in the groups LIP, AA/NT/O, DTOX, MIT, GLY, OTH, and RDX. (D) Examples of expression patterns in the wing disc for different members of the Metabolism class (name of each gene in the bottom-left corner of each picture). Most genes are expressed in a ubiquitous manner in the wing disc. *In situ* hybridizations pictures correspond to experiments published as supplementary material in Molnar *et al.* (2012), Organista *et al.* (2015), and Hevia *et al.* (2017). (E) Representative wings of knockdowns of Metabolism genes belonging, from top to bottom, to the AA/NT (top), DTOX, GLY, and LIP (bottom) classes. Wings corresponding to *Lsd*-*2*, *Gbs-70E*, and *Hmgcr* are from *UAS-Dicer2/+; sal^EPv^-Gal4/UAS-RNAi* flies and the remaining from *UAS-Dicer2/+; nub-Gal4/UAS-RNAi* flies. The name of each gene is given in the bottom-left corner of each wing picture.

In “Metabolism” we include all proteins involved in the synthesis and degradation of lipids, sugars, nucleotides, and aminoacids (anabolism and catabolism) and also proteins involved in detoxification, xenobiotic processing, autophagy, and maintenance of the RedOx balance. We also included in this class all genes related to mitochondrial biology. The metabolism class is large (1631 genes; Figure 7A and Supplementary Table S1.7) and was subdivided into several groups: “Amino acids, Nucleotides and other related molecules” (MET-AA/NT/O; 278 genes), including the biosynthesis of amino acids and molecules derived from amino acids, biosynthesis of purine and pyrimidine nucleotides and molecules derived from nucleotides and Nitrogen metabolism; Oxido-reduction metabolism (MET-RDX; 94 genes); “Carbohydrates and glycobiology” (MET-GLY; 200 genes), including disaccharides and polysaccharides digestion, cellular transport of monosaccharides, Glycolysis and gluconeogenesis pathways, Pyruvate degradation, pentose phosphate pathway, and Glycoconjugates metabolism; Lipid metabolism (MET-LIP, 489 genes) including Lipid transport, fatty acids biosynthesis, triacyclglycerols metabolism, membrane phospholipids metabolism and cholesterol, steroid and isoprenoids metabolism; “Detoxification” (MET-DTOX; 208 genes), including Cytochromes involved in oxide-reduction reactions, oxidative stress and detoxification of endogenous and xenobiotic compounds, central metabolism, including tricarboxylic acid cycle (MET-CTR; 38 genes), and “Mitochondrial biology” (MET-MIT; 207 genes), including respiratory chain complexes components, electron carriers, and other mitochondrial proteins (Supplementary Table S1.7; Figure 7A). The remaining 155 genes (“MET-OTH”) were classified into four additional groups: “Autophagy,” including peptidases related with autophagy and proteins related to autophagosome assembly (MET-ATG; 27 genes); “One-carbon metabolism” (MET-OC; 13 genes); “Metal metabolism” (MET-MTA; 35 genes); and “Xenobiotic metabolism” (MET-XEN, 42 genes).

Within the MET class, we found expression in the wing imaginal disc in the range of 55–82% of genes (Figure 7B), and a low percentage of genes resulting in a mutant phenotype in knockdown conditions (28% in average), with the exception of genes involved in mitochondrial biology, for which this percentage raises to 54% (Figure 7B). Mutant phenotypes were similarly distributed in all different subgroups (Figure 7C), with a greater prevalence of pupal lethal phenotypes in the mitochondrial biology group (Figure 7C). For members of the different groups included in the Metabolism class, we found a range of visible phenotypes that were also observed in other molecular classes (Figure 7E). In some cases, these phenotypes were reminiscent of Notch signaling insufficiency (Lipid class; Figure 7E). In general, the expression of these genes visualized by *in situ* hybridization was ubiquitous in the wing disc (Figure 7D).

### Cuticle

**Figure 8:**
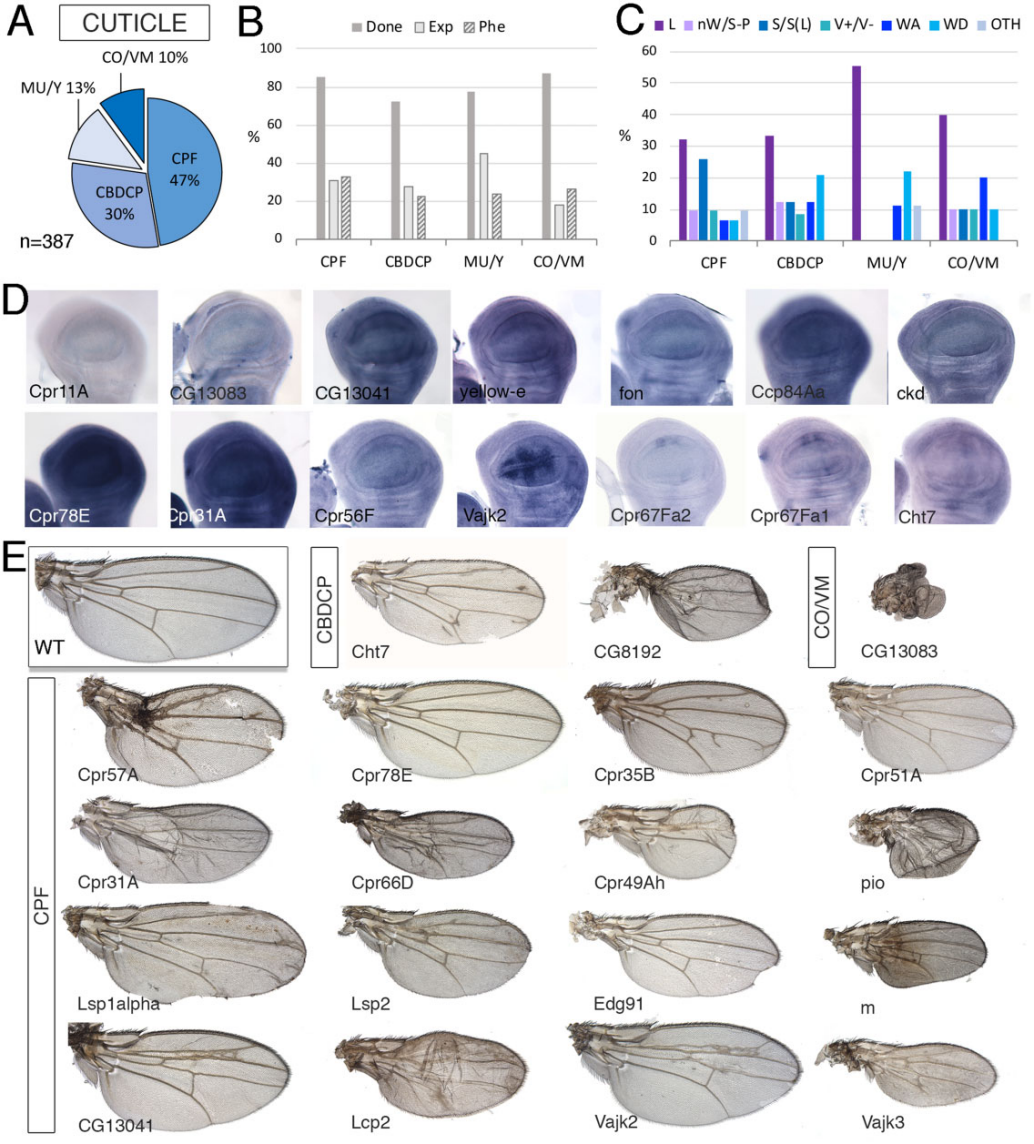
Cuticle class. (A) Distribution of the 387 genes included in the “Cuticle” class into the groups “Cuticular Protein Families” (CPF), “Chitin binding domain containing proteins” (CBDCP), Mucins, Glue proteins, Chitin deacetylase-like family and yellow family (MU/Y), and Chorion and vitelline membrane proteins (CO/VM). (B) Percentages of genes within each group for which we tested the knockdown phenotype (Done; dark gray column), of genes expressed in the wing disc (Exp; light gray column), and of genes with a lethal or visible phenotype in knockdown conditions (Phe; striped column). (C) Frequency of lethal (L) and visible mutant phenotypes observed in the CPF, CBDCP, Mu/Y, and CO/VM groups. Note the high frequency of “Size” phenotypes in the CPF group. (D) Examples of expression patterns in the wing disc for different members of the Cuticle class (name of each gene in the bottom-left corner of each picture). *In situ* hybridizations pictures correspond to experiments published as supplementary material in Molnar *et al.* (2012), Organista *et al.* (2015), and Hevia *et al.* (2017). (E) Representative wings of knockdowns of Cuticle genes belonging, from top to bottom, to the CBDCP, CO/VM, and CPF groups. All wings correspond to *UAS-Dicer2/+; nub-Gal4/UAS-RNAi* flies. The name of each gene is given in the bottom-left corner of each wing picture.

We include all structural and related constituents of external membranes such as the adult cuticle, eggshell, and gut peritrophic matrix (Figure 8). The “Cuticle” group includes 387 genes of which only 30% are expressed in the wing disc (Figure 8). This group was further divided into four groups (Figure 8A): “Cuticular Protein Families,” which are the structural proteins of the adult cuticle (CUT-CPF; 183 genes), “Chitin binding domain containing proteins” (CUT-CBDCP; 116 genes), Chorion and vitelline membrane proteins (CUT-CO and CUT-VM; 39 genes), and a fourth group including the Mucins, Glue proteins, and yellow family (CUT-MU and CUT-Y; 49 genes). As expected, these groups have low frequencies of genes expressed in the wing disc (from 18% for CUT-COP/CUT-VM to 45% for CUT-MU/CUT-Y genes; Figure 8B), and low frequencies of genes with a knockdown causing lethality (from 9% for CUT-CBDCP to 13% for CUT-MU/CUT-Y genes; Figure 8, B and C) or a mutant phenotype (from 10% for CUT-MU and CUT-Y to 26% for CUT-CPF genes; Figure 8, B and C). For genes of this class expressed in the wing disc, we found ubiquitous expression and also cases of patterned expression related to the developing wing veins (Figure 8D). Most visible phenotypes correspond to genes belonging to the CUT-CPF group (59% of described phenotypes; examples in Figure 8E), which were very prominent in phenotypes affecting the size of the wing (Figure 8, C and E). The groups CUT-CBDCP and CUT-MU/CUT-Y exhibit wing differentiation phenotypes with frequencies higher than other cuticle genes (Figure 8C and Supplementary Figure S1B).

### CG and CGh

**Figure 9:**
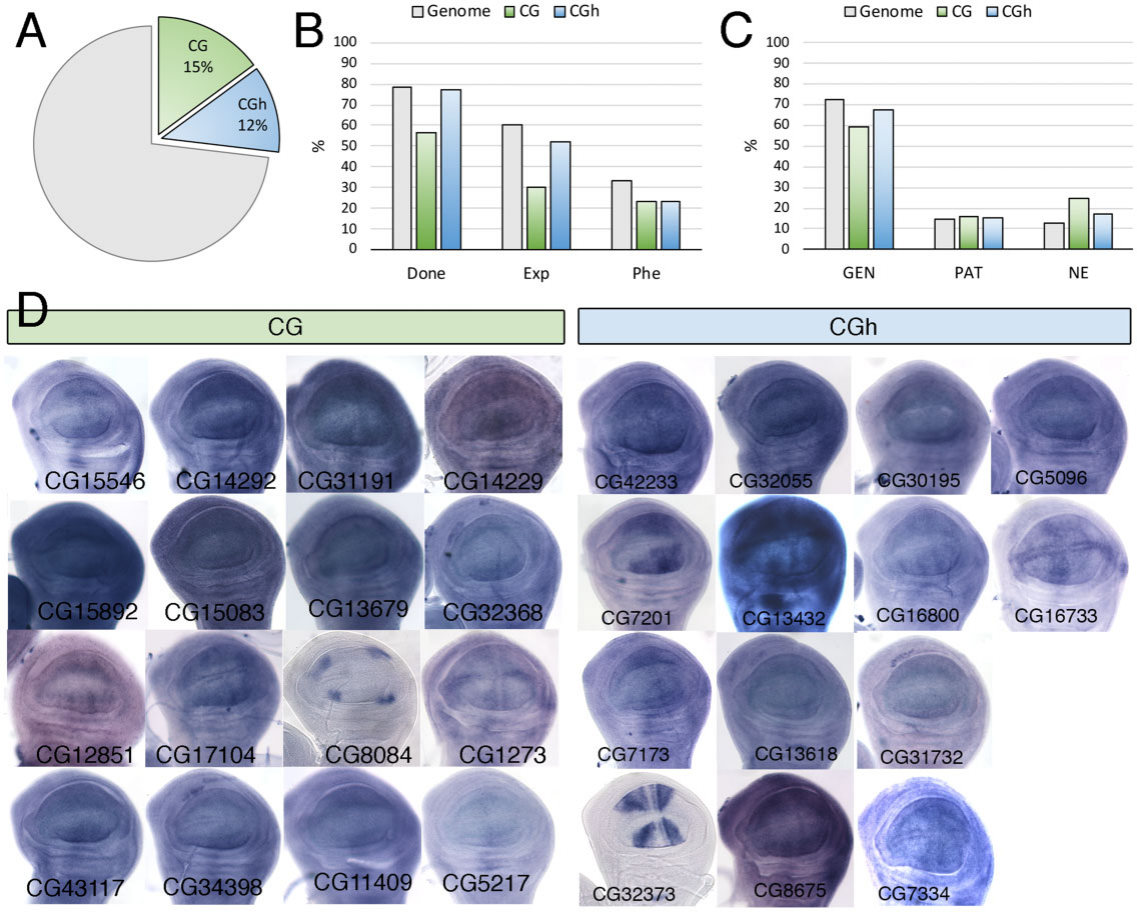
Expression analysis of the CG and CGh classes. (A) Percentage in the genome of members of the functional classes CG (CG, green sector, 2084 genes) and CGh (CGh, blue sector, 1675 genes). (B) Percentage of CG (green columns) and CGh (blue columns) genes analyzed in knockdown conditions (Done), expressed in the wing disc (Exp), and resulting in lethality or a mutant phenotype (Phe). The same values are indicated as a reference for the entire genome (gray columns). (C) Percentage of CG (CG, green column, 44 genes) and CGh genes (CGh, blue columns, 58 genes), and all functional classes grouped together (Genome, gray columns; 562 genes) expressed in a ubiquitous manner (GEN), not expressed (NE), and expressed in a spatial pattern (PAT). (D) Examples of expression patterns in the wing disc for different members of the CG and CGh classes. The name of each gene is indicated in the bottom-left corner of each picture. *In situ* hybridizations pictures correspond to experiments published as supplementary material in Molnar *et al.* (2012), Organista *et al.* (2015), and Hevia *et al.* (2017).

**Figure 10:**
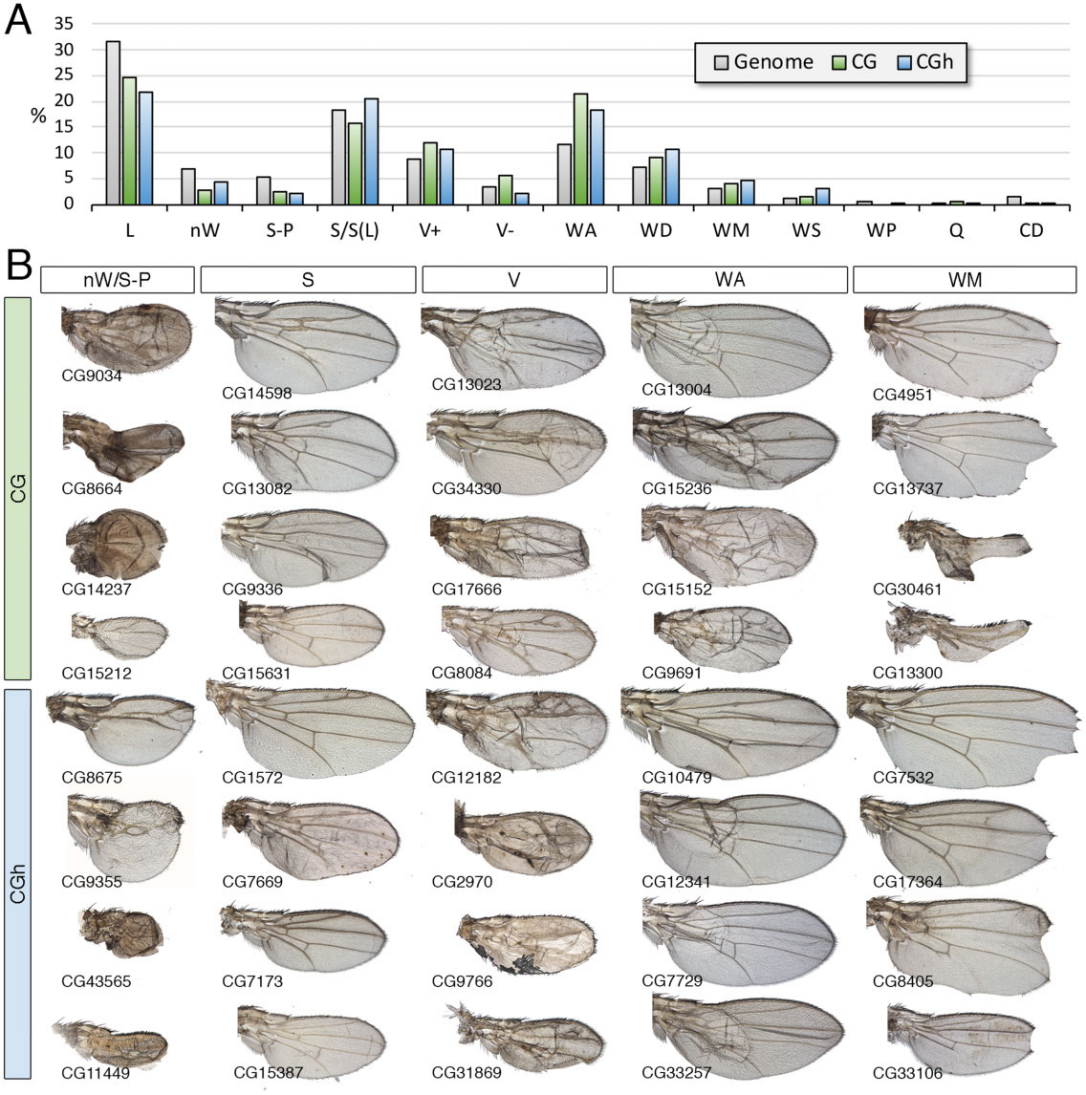
Phenotypic analysis of the CG and CGh classes. (A) Frequency of knockdown phenotypes for genes belonging to the CG class (green columns) and CGh class (blue columns) compared to the genome (gray columns). Lethal combinations (lethal), lack of wing (nW) defects in wing size and pattern (S-P), defects in wing size (S), differentiation of ectopic or thicker veins (V+), loss of veins (V−), appearance of wing blisters (WA), wing differentiation defects (WD), loss of wing margin structures (WM), alterations in wing shape (WS), defects in cuticle pigmentation (WP), appearance of extra bristles in the wing surface or loss of bristles in the wing margin (Q) and defects in trichome differentiation and cell size (CD). (B) Representative wings of knockdowns of CG (upper rows, green code) and CGh (lower rows, blue code) belonging, from left to right, to the phenotypic classes nW/S-P, S, V (V+ and V−), wing adhesion (WA) and wing margin (WM). Wings corresponding to CG8675 and *dy* are from *UAS-Dicer2/+; sal^EPv^-Gal4/UAS-RNAi* flies, the remaining wings from *UAS-Dicer2/+; nub-Gal4/UAS-RNAi flies*. The name of each particular gene is given in the bottom-left corner of each wing picture.

The CG class, containing genes for which there is a complete lack of functional information, and the CGh class including genes with at least one InterPro (IP) domain, are among the most abundant in the Genome, including 2084 (CG) and 1675 (CGh) genes (Supplementary Table S1). The most frequently found conserved domains in the CGh class, found in more than 20 genes, were “Protein of unknown function DUF1091” (IPR010512; 91 genes), “Leucine-rich repeat” (IPR001611; 35 genes), “Protein of unknown function DM4/12” (IPR006631; 30 genes), “Haemolymph juvenile hormone binding” (IPR010562; 26 genes), “EF-hand domain pair” (IPR011992; 23 genes), and “Alkaline-phosphatase-like, Core domain superfamily” (IPR017850; 22 genes). The CG and CGh genes include 15% and 12% of the fly genome (Figure 9A), but only a small fraction of CG genes (30%) were considered as expressed in the wing disc (Figure 9B). For those genes that were expressed, the more prevalent type of spatial pattern visualized by *in situ* hybridization was ubiquitous (Figure 9, C and D), but many instances of genes expressed in restricted patterns were also identified (Figure 9, C and D). The frequency of different phenotypes we found in the CG and CGh classes were similar to each other (Figure 10A). For most phenotypes, we found a lower frequency compared with the genome, the only remarkable exception being defects in dorsoventral adhesion (WA) (Figure 10, A and B). Many phenotypes affecting the formation of the veins and wing margin, as well as reductions in the size of the wing (Figure 10B), suggest that numerous *Drosophila* genes without clear orthologs in other noninsect species play important roles during wing development.

## Concluding remarks

We present here a simplified classification of *Drosophila* protein-coding genes that aims to contain in only two terms the main features of each protein. We have used this classification to incorporate data from mRNA expression and from the results of an RNAi phenotypic screen in the wing. As expected, no functional class or group within a class could be subscribed to a particular phenotype. However, some phenotypes are enriched in particular functional classes, including cell viability phenotypes in the Ribosome, Protein transport and RNA classes, wing vein patterning in the Cell signaling class and failures in dorsoventral apposition in the cell adhesion class (Supplementary Figure S1). We also present a phenotypic description for a large set of genes for which there is very little functional information (CG and CGh classes). Although these phenotypes are not predictive of molecular or biological function in the adult *Drosophila* wing, we expect that they open new entry points to undertake the functional analysis of the corresponding genes.

## Data availability

The data underlying this article are available in the article and in its online supplementary material. All wing pictures we have were submitted to the Figshare repository: https://doi.org/10.6084/m9.figshare.16624645.v1; https://doi.org/10.6084/m9.figshare.16624630.v1; https://doi.org/10.6084/m9.figshare.16624603.v1; and https://doi.org/10.6084/m9.figshare.16624591.v1

## Supplementary Material

jkab349_Supplementary_Data
