# Supplementary material for: Genome-wide phenotypic RNAi screen in the Drosophila wing: phenotypic description of functional classes
Source: G3 (Bethesda). 2021 Oct 2;11(12):jkab349. doi: 10.1093/g3journal/jkab349 (PMC8664486; doi:10.1093/g3journal/jkab349)
Supplement: jkab349_Supplementary_Data [file jkab349_supplementary_data.zip › GENETICS-G3-2021-402594-s01.docx]

**Legends to Supplementary Figure and Tables**

**Supplementary Figure 1**

(A) Relative contribution of each of 14 functional groups to a specific phenotypic class, ranging from Cell Adhesion (CA), DNA Biology (DNA), Metabolism (Met), Protein Biology (PRO), Ribosomal biology (RIB), RNA Biology (RNA), Signaling (SIG) and Transport across membranes (TRA), Cuticle proteins (CUT), Cytoskeleton (CYT), Protein transport across membranes (PTR), Cell Division (DIV), CG and CGh groups. (B) Relative contribution of each subgroup to a specific phenotypic class within a functional category. From upper to bottom: Cell Adhesion, DNA, Metabolism, Protein, Ribosome, RNA, Signaling and Transport classes. Red dots at the “Y” axis of the graphs points the expected frequency value for each group. For frequency distribution analyses, Chi-square tests were performed for each phenotype and p-values of >0,05 (*), >0,01 (**) and >0,001 (***) were considered statistically significant.

**Supplementary Table 1**

**Distribution of all *Drosophila* genes into 16 functional classes**

RNAi strain (TransformantID), Flybase identifiers (FlybaseID), Gene names (CGNR and Gene symbol), Phenotypic description (Phenotype) using the abbreviations described in Table 1. MN column: Molecular classes as defined in the text and Table 1. Expression Affi: Average expression level in Affimetrix microarray, Expression RNAs: tpm in RNAseq data. Expression: Positive (Y) and negative (N) expression in the wing disc.

**Supplementary Table 2**

List of phenotype and molecular class/group abbreviations in MS Exel format

**Supplementary Table 3**

Localization in the Figures and Supplementary Figures of wing pictures and *in situ* hybridization wing disc from this manuscript and from López-Varea *et al*., 2021a and Ostalé *et al*., 2021.
