# Supplementary figures and images for: Genome-wide phenotypic RNAi screen in the Drosophila wing: phenotypic description of functional classes
Source: G3 (Bethesda). 2021 Oct 2;11(12):jkab349. doi: 10.1093/g3journal/jkab349 (PMC8664486; doi:10.1093/g3journal/jkab349)

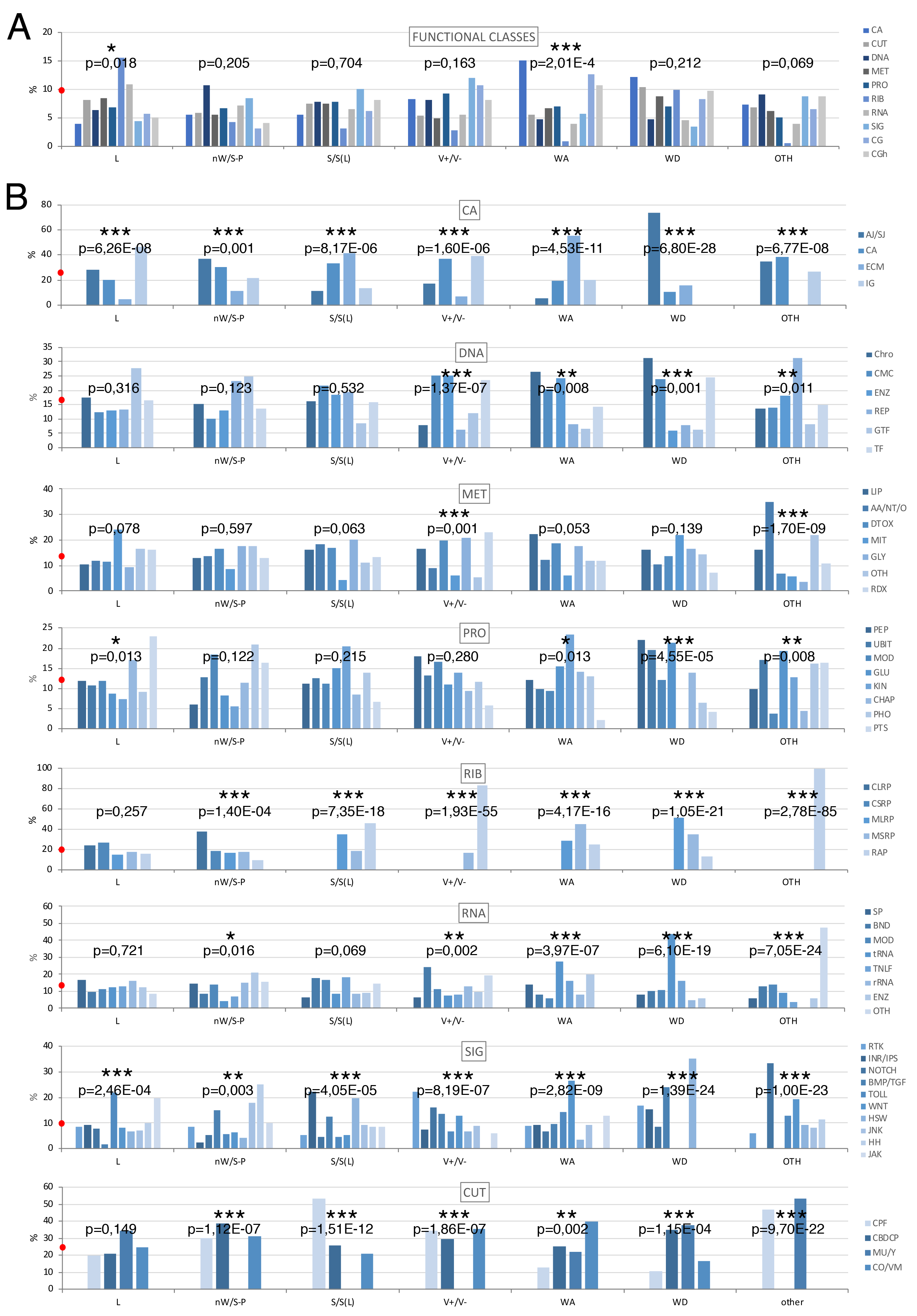

Supplement: jkab349_Supplementary_Data [file jkab349_supplementary_data.zip › GENETICS-G3-2021-402594-s02.tif]
